# Shoulder Evaluation by Telephone and Video Visit: A Narrative Review

**DOI:** 10.7759/cureus.22461

**Published:** 2022-02-21

**Authors:** Andres I Applewhite, Robert Gallo, Matthew L Silvis, Ashley L Yenior, Angie N Ton, Cedric J Ortiguera, George Pujalte

**Affiliations:** 1 Department of Internal Medicine, Mayo Clinic, Jacksonville, USA; 2 Department of Orthopaedics and Rehabilitation, Penn State Health Milton S. Hershey Medical Center, Hershey, USA; 3 Department of Family Medicine, Penn State University, Hershey, USA; 4 Department of Family Medicine, Mayo Clinic, Jacksonville, USA; 5 Department of Family Medicine, Mayo Clinic, Scottsdale, USA; 6 Department of Orthopaedic Surgery, Mayo Clinic, Jacksonville, USA

**Keywords:** musculoskeletal injury, orthopedic practice, shoulder pathology, telehealth education, telemedicine (tm)

## Abstract

Telemedicine has a very important role in today’s healthcare system, which has been accentuated during the SARS-CoV-2 pandemic. Virtual medical evaluations offer a myriad of benefits for both patients and providers. Evaluations of the musculoskeletal system, however, present unique challenges because diagnosis significantly relies on a physical examination, something not easily accomplished by virtual means. The shoulder, a complex region with four separate articulations, is no exception. Nevertheless, a properly planned and executed telemedicine visit may yield successful results even with challenging shoulder pathologies. This narrative review aims to offer clinicians who are novices in the practice of telemedicine a basic framework with instructions, questions, and some examples of interpretation of patient answers to guide them through encounters for the evaluation of shoulder complaints via telephone and video consultation.

## Introduction and background

Stay-at-home and social distancing measures implemented during the SARS-CoV-2 pandemic highlighted the need to improve access to medical care for patients unable to see their physicians in person [1,2]. Some people avoid healthcare facilities for multiple reasons, such as fear of germ exposure or difficulty in transportation, and therefore unnecessarily miss or defer medical appointments. Also, some metropolitan areas have overburdened healthcare systems, and obtaining medical appointments may result in weeks or months of waiting time; moreover, many pathologic conditions, if not addressed promptly, may lead to poor outcomes. Direct telephone and video evaluations are a safe, productive, and convenient alternative to ensure people continue to receive medical care when in-person visits are not possible [3-5]. As technology and audiovisual devices improve and become more readily available and user-friendly, the benefits of telemedicine become clearer. 

Some studies suggest that virtual medical visits may have similar health outcomes and patient satisfaction to traditional in-person visits. A randomized controlled trial by Buvik et al. involving 389 patients divided the study population into two groups, traditional face-to-face orthopedic consultation and video-assisted telemedicine, and compared health outcomes and patient satisfaction measured with self-reported questionnaires [6]. They found no statistically significant difference in patient satisfaction or health outcomes measured 12 months after the consultations, suggesting that video-assisted telemedicine orthopedic consultation can be as satisfactory for the patient as a traditional in-person medical visit. Furthermore, 86% of the patients receiving video-assisted consultations expressed they would choose the same telemedicine method as their next medical visit [6]. Results from this study suggest that most people may prefer virtual medical visits as their primary method of consultation while maintaining similar health outcomes. This interesting finding highlights the importance of universally offering patients the option of virtual medical visits.

Evaluating musculoskeletal conditions through telemedicine presents unique challenges because physical examinations are a key component of diagnosis and are not easily achieved through virtual means. Shoulder evaluation is no exception. The shoulder, with four separate articulations and six directions of freedom, is the most mobile joint in the body; however, this tremendous degree of mobility comes with a vulnerability to injury [7]. Shoulder ailments may be debilitating and can lead to pain and suffering. They may also worsen symptoms of depression or cause patients to miss work [8]. In addition to pain stemming from intrinsic shoulder pathology, other concerning conditions such as myocardial ischemic events, cholelithiasis [9], and herniated cervical intervertebral discs may often manifest as “shoulder pain” and, if not recognized promptly, may result in permanent disability, dysfunction, or death [10]. These conditions may be recognized during a telemedicine encounter and proper steps to prevent further complications may then be taken. 

Despite the benefits of remote visits, some patients may be reluctant to engage in telemedicine due to multiple reasons [11]. To maximize the benefits of virtual consultations, physicians must have a thorough understanding of shoulder anatomy, mechanics behind injuries, classical findings during evaluations, and be familiar with current treatment and management guidelines. Physicians must also develop a mental framework for guiding patients to adequately describe their symptoms and perform physical self-evaluations. This article can be a reference guide for clinicians who are novices in the practice of telemedicine for the evaluation of shoulder complaints. 

## Review

Preparation for virtual encounters

Telemedicine visits rely on the quality of the technology used. Voice clarity, image quality, patient preparation (including proper attire), and patient positioning are crucial for conducting an adequate virtual physical examination. During a video encounter, the patient should be in a well-lit room with the light source facing the patient (not coming from behind) and the camera should not be facing mirrors or windows with bright lights, as this may cause excessive backlighting and obscure details [12]. Patients should position the camera on a steady surface, at a distance of approximately six feet away (different cameras have different features) and at a height of at least 4-5 feet off the ground (e.g., on a table, counter, or stairs) with enough room to extend arms in all directions [12]. In a telephone encounter, it is ideal for the patient to have a large mirror in lieu of the camera, so they may self-inspect during the physical examination, and describe what they see to the physician. Patients should wear a tank top to expose their shoulder joints. A sturdy chair or stool with a comfortable height should be used. Some provocative tests (see ahead) are ideally executed with the patient lying down flat on the side of a stable bed or couch.

Items of known weight appropriate for each patient (small dumbbell, can of vegetables, a container with water, etc.) may be used to simulate resistance for the physical examination segment of the encounter. Fragile or glass containers are discouraged due to the risk of being dropped and broken. These requirements should be made clear to the patient a few days prior to the interview so that there is ample time to prepare. For video encounters, a screen-sharing function may be helpful to allow the physician to show surface anatomy and pain diagrams to the patient. 

Shoulder evaluation by telephone

Shoulder evaluation by audio-only (i.e. by telephone), without the benefit of video, is likely to be more challenging. However, a carefully carried-out telephone consultation when visualization by video is not possible may still have a positive outcome for patients. In cases when a patient does not have access to a healthcare facility and is visually impaired, lacks the technology or understanding of video platforms, has inadequate lighting or poor video conditions, or is trying to get first access during a crisis situation, a physician may still be able to obtain important information to evaluate shoulder complaints using a telephone visit.

As with any medical consultation, the first step in a telephone visit is to take an exhaustive and meticulous patient history to formulate an initial differential diagnosis. Specific details regarding the main complaint, medical, surgical, and social history, previous injuries, current medications, jobs, hobbies, and activities, and a review of each organ system, should be carefully obtained [13]. The clinician must be able to verbally guide patients through certain actions to explore a range of motion, pain, visual and tactile features, etc., in order to formulate questions that elicit detailed responses. The clinician should be mindful about choosing words that patients understand and reformulate questions when they provide unclear answers. It is also important for the patient to have a large enough mirror to see their limbs during self-inspection. 

The patient’s age can help narrow down this list. For example, a young patient with a traumatic event who feels their shoulder could “come out” (as a subluxation or dislocation is commonly described) can be deemed to have shoulder instability, in which case, imaging, in-person visit, or physical therapy referral may be warranted. In contrast, a middle-aged woman with a thyroid disorder and diabetes who has a gradual onset of shoulder pain and stiffness might be suffering from adhesive capsulitis. In an older patient with sharp pain who has just suffered trauma to the shoulder, a sudden inability to raise the arm over the head would raise concern for dislocation or fracture, requiring urgent imaging and in-person follow-up [14]. If, however, an older patient suffers from gradual progressive weakness and pain while reaching overhead, a rotator cuff pathology or impingement syndrome may be suspected, which can be managed conservatively without imaging. Providers should also remember to specifically ask questions regarding the cervical spine during assessment because referred pain is commonly misinterpreted by patients as primary shoulder pain.

Physicians may also ask the patient to take pictures of any outstanding findings during the self-inspection with a cellular telephone camera, perhaps with the help of a family member or friend, and ask them to send the pictures to an office smartphone or email. This would allow the physician to quickly visualize and evaluate anything the patient finds striking, which may afford important clues to the overall condition.

**Figure 1:**
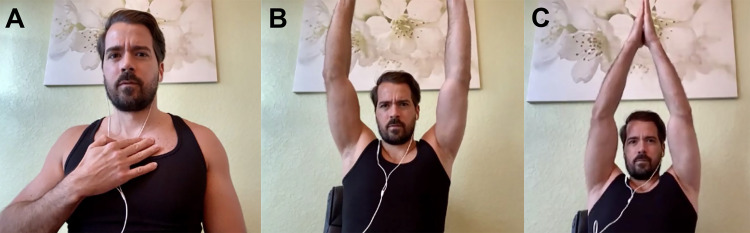
A: Palpation of Clavicular Region. B: Abduction of Arms. C: Overhead Arm Clap.

**Figure 2:**
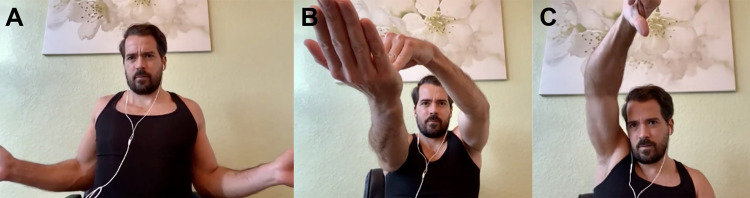
A: External Rotation of Arm. B: Modified Speed’s Test. C: Modified Empty Can (Job’s) Test.

**Figure 3:**
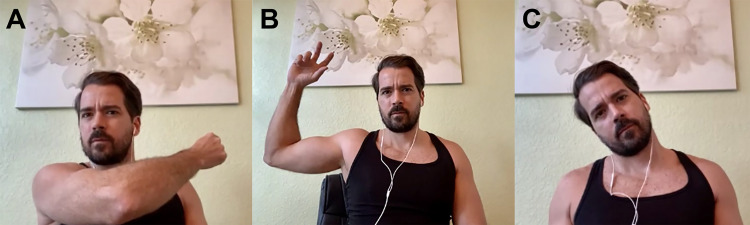
A: Modified Crossover (Scarf) Test. B: Modified Apprehension Test. C: Assessment of Cervical Spine Range of Motion.

Table 1, Figures 1A-1C, and Figure 2A provide questions and instructions that may be formulated by clinicians via telephone and possible implications of the responses [15-19]. Table 2, Figures 2B-2C, and Figures 3A-3C provide questions and instructions for common provocative shoulder tests [18,20-25]. It is up to the physicians to determine whether they feel confident enough with patients following these instructions in an accurate manner to gain reliable information from provocative tests.

**Table 1:** Shoulder Evaluation by Telephone – Questions and Instructions *Figure 1A; **Figure 1B; ***Figure 1C; ¤Figure 2A

What to say to the patient during a telephone encounter	Possible implications
“Stand in front of a mirror and inspect both exposed shoulders, front, and back. Compare them. Do you see any striking differences?”	Evaluate asymmetry from atrophy, swelling, ecchymosis or erythema, deformity, scars, or venous distension. These may have a lot of implications that should be taken into consideration with the rest of the examination.
“Looking at your shoulders in a mirror, do you see any difference in height between your left and right shoulders?”	Striking differences in shoulder height may suggest paraspinal muscle spasm from cervical spine pathology, nerve injury (such as spinal accessory nerve), guarding from massive rotator cuff tear, mass, acromioclavicular separation, or degenerative changes [16].
“If you can see the back of your shoulders in a mirror (perhaps with a second hand-held mirror), and trying to keep a neutral position on both sides, are you able to see a striking difference between them? Has anyone commented that your shoulders look different from each other when viewed from the back?”	Striking differences in shoulder prominence from posterior view may suggest nerve injury (such as spinal accessory, dorsal scapular, or long thoracic nerve), muscle atrophy from chronic massive rotator cuff tear, sick scapular syndrome, nerve entrapment (suprascapular nerve due to paralabral cyst), brachial neuritis, iatrogenic injury, cervical radiculopathy [17].
“Have you or anyone else noticed any sunken, swollen, bruised, and/or red areas on your shoulder?”	Sunken areas could represent atrophied areas. Swollen or bruised areas may represent contusions, fractures, or tendon ruptures. Red areas may indicate infection [17].
“Does it hurt to press anywhere along your collarbone (starting from the chest all the way to the shoulder)? Do you feel any bubbles under your skin or general deformities?” *	Pain at any of these sites may have a lot of implications, such as sternoclavicular or acromioclavicular instability, sprain, dislocation or arthritis [15], clavicle fracture (potentially displaced if crepitus or deformity appreciated), rib fracture, supraclavicular nerve contusion, distal clavicle osteolysis [19].
“Does it hurt to press on the front of your shoulder?”	This may suggest long head of bicep tendinopathy or instability, subscapularis tendon tear, pectoralis major rupture or strain, glenohumeral arthritis, adhesive capsulitis, anteroinferior labral tear, glenoid or proximal humerus fracture, Salter-Harris fracture in adolescents [18].
“Does it hurt to press on the side of your shoulder, where the bony part on top of it ends?”	This may suggest supraspinatus tendinopathy or tear, calcific tendinitis, acromial fracture, Salter-Harris fracture in adolescents, deltoid tear or strain (less likely) [18].
“Starting with your arms hanging down at your sides, can you reach out in front of you, then upwards towards the ceiling with both arms?” **	If pain and/or weakness is experienced, this may suggest subacromial impingement or supraspinatus pathology; cervical radiculopathy; proximal humerus or clavicle fracture; adhesive capsulitis; glenoid labrum tear; acromioclavicular joint sprain; glenohumeral arthritis; deltoid, pectoralis, or coracobrachialis tear or strain (less likely) [18].
“Starting with your arms hanging down at your sides, can you reach backwards then upwards with both arms?”	If pain and/or weakness is experienced, this may suggest adhesive capsulitis, latissimus dorsi, subscapularis tendon, or deltoid tear or strain (less common) [17].
“Starting with your arms hanging down at your sides, can you reach out to your sides then upwards with both arms? Are you able to clap your hands directly above your head?” ***	If pain and/or weakness is experienced, this may suggest supraspinatus tendon tear or tendinopathy; subacromial bursitis; cervical radiculopathy; nerve injury (such as spinal accessory, dorsal scapular, or long thoracic nerve), brachial neuritis; adhesive capsulitis; proximal humerus, acromion, or clavicle fracture; deltoid strain (less common) [18].
“Starting with your arms hanging down at your sides, bend your elbows to 90⁰ with hands in front of you. Keeping your elbows touching your sides, can you swing your hands out to the sides away from each other?” ¤	If pain and/or weakness is experienced, this may suggest glenohumeral arthritis, adhesive capsulitis, proximal humerus fracture [17].
“What tasks have you found difficult to execute due to weakness, or cause pain or range-of-motion limitation of the shoulder?” For strength testing, the clinician may also ask the patient to reproduce tasks/movements/exercises over the telephone and describe weakness and/or pain felt.	This gives the patient an opportunity to express any specific concern they may have in mind. The responses may give clues to strength problems in the shoulder. Depending on the description of the tasks causing weakness, some common pathological culprits could be the rotator cuff, biceps, or deltoid [17].
Ask patient: “Do you have any pain that runs down your arm past the elbow?”	An affirmative response is concerning for a cervical nerve root pathology [16].

**Table 2:** Provocative Tests for Shoulder Evaluation via Telephone or Video Visits Note: Tests such as the posterior apprehension test, sulcus sign test, labrum grind test, clunk test, or relocation test (which explore for shoulder instability and labrum tears); as well as maneuvers for cervical spine pathology, should only be done by an experienced physician during in-person visits. These tests are not recommended to be carried out over video or telephone consultations. *Figure 2B; **Figure 2C; ¤Figure 3A; ¤¤Figure 3B; ¤¤¤Figure 3C

These classical tests may be modified by asking the patient to carry objects of known weight (such as a light dumbbell, water bottle, a bean can, pasta sauce bottle, etc.) in lieu of examiners active resistance to movements. Glass containers should be avoided for they are prone to causing accidents if dropped.	
Apley Scratch Test “With the affected arm, going behind your head, attempt to touch your back over the scapula of the opposite side (abduction & external rotation). Now try to touch the same spot with the same hand but going behind your lower back instead (internal rotation and adduction). Repeat the same movements with the opposite arm to compare.”	Loss of range of motion could represent a rotator cuff pathology.
Speed’s Test (modified) “With the affected arm outstretched in front with a 15° bend in the elbow, place hands palms-up (supination) while holding a weighted object and slowly raise the as far up as possible starting from waist level” Alternatively, the patient may push down on affected hand with other side and try to resist this movement.* Sensitivity: 32% [23] Specificity: 61% [23]	Pain in the anterior shoulder (site of the long head of biceps tendon insertion) is considered a positive test and may indicate a lesion in the biceps tendon or labrum pathology.
Yergason’s Test (modified) “Place the affected arm against the side of your thorax, then bend your elbow 90° and turn the hands palms-down (pronation). With the unaffected handhold the affected hand, and while keeping arm tight against thorax, resist the following three movements: twisting the forearm towards palms up (supination), flexion of the forearm over the arm, and swinging forearm outward (external rotation of humerus).” Sensitivity: 43% [24] Specificity: 79% [24]	Pain at the superior glenohumeral joint suggests a superior labrum anterior-posterior (SLAP) lesion or pathology in the long head of biceps tendon [18]. Note: normally, the examiner palpates the bicipital groove for a snap which would suggest a tear or laxity of the transverse humeral ligament, but unfortunately this cannot be explored by the patient while performing this modified test.
Empty Can (Job’s) Test (modified) “Grab a weighted object with the hand of the affected side, stretch arm out in front of you with 30° of flexion in your elbow, and twist your forearm until thumb is pointed down (internal rotation), and hold this position for 3 seconds if possible.” ** Sensitivity: 52.6% [22] Specificity: 82.4% [22]	The test is considered positive if weakness or pain is experienced in the shoulder by the patient, and it suggests a lesion in the supraspinatus tendon, rotator cuff impingement, or neuropathy of the suprascapular nerve [18,25].
Full Can (Neer) Test (modified) This test is very similar to the Empty Can test, except for the thumb is now pointing up toward the roof (instead of down toward the floor). Sensitivity: 79% [23] Specificity: 53% [23]	(same as above)
Hawkins-Kennedy Test (modified) “Grab a weighted object with the hand of the affected side, flex shoulder to 90° and flexes the elbow 90° so that forearm is in front of you horizontally. From this position, slowly swing forearm down towards the floor (internal rotation).” Sensitivity: 79% [23] Specificity: 59% [23]	This test is considered positive if the patient experiences shoulder pain during the maneuver, and it may suggest subacromial impingement or rotator cuff tendinopathy [18,25].
Crossover (Scarf) Test (modified) “Place arm of affected shoulder straight out in front of you (shoulder flexed at 90°). With opposite hand, grab the elbow of the affected side and pull the affected arm towards your chest horizontally (as if you were placing a scarf around your neck) until full range of motion.” ¤ Sensitivity: 77% [20] Specificity: 79% [20]	This test is considered positive if the patient experiences shoulder pain during the maneuver, and it may suggest pectoralis major or subscapularis tendon tear, acromioclavicular joint pathology, posterior labral tear, clavicle or proximal humerus fracture [18,25].
O’Brien Test (modified) This maneuver will have two parts to it. “Grab a weighted object in each hand, straighten your arms in front of you (90⁰ of shoulder flexion), and bring your hands close together – about 3 inches apart (10º-20º horizontal adduction). From this position, twist your forearms inward until thumbs point down towards the floor (full pronation) and hold this position for three seconds.” “Afterwards, from the previous position, twist your forearms outward until palms are facing up towards ceiling (full supination) and hold this position for 3 seconds.” (Sensitivity and specificity reports vary widely in literature)	This test is considered positive if the pain is elicited during the first maneuver (thumbs facing down) and reduced/eliminated during the second maneuver (palms facing up). It suggests labral pathology. If pain is experienced with both maneuvers, it suggests AC joint pathology.
Apprehension Test (modified) “If possible, lay down flat on your back on a couch or bed, with the arm of the affected side on the edge. Grab a weighted object with the hand of the affected side, and slowly flex your shoulder at 90º and elbow at 90º as if you were going to throw a baseball. Try to reach the end of the range of motion.” ¤¤ (however it is best to perform in supine position) Sensitivity: 53% [22] Specificity: 99% [22]	The test is considered positive if the patient feels apprehension (feeling of instability or that shoulder is going to “pop-out” or dislocate), and this would suggest instability of the glenohumeral joint in an anterior direction. If the patient feels pain with this maneuver instead of apprehension, a different pathology (such as rotator cuff impingement or glenohumeral arthritis) may be present [18,25].
The cervical spine should be assessed as a possible etiology for shoulder pain. Ask the patient to palpate his/her cervical spine for areas of tenderness. Also assess flexion, extension, lateral rotation and bend by asking the patient: “Look up, down, and to the sides. Bend your neck so that your left/right ear touches your left/right shoulder respectively. Place the palm of the hand of your affected side on top of your head, does this relieve the pain? (Shoulder Abduction Relief Sign Test).” ¤¤¤ Note: virtual evaluation of cervical spine is limited. If cervical spine pathology is suspected, the patient should be advised to schedule an in-person.	Cervical spine pathology (such as radiculopathy, arthritis, sprain/strain, or fracture) may be the source of shoulder pain if the patient experiences pain or limitation with flexion, extension, twisting, or side bending; or tenderness on spinous processes or paraspinal muscles [16]. Relief of pain during shoulder abduction relief sign test suggests cervical radiculopathy, especially when lower cervical roots are involved.

By considering patients’ detailed history and information gained through questions and self-physical examination, it is possible to exclude emergencies and formulate a feasible differential diagnosis for shoulder conditions via telephone. Table 3 provides sample notes for findings during a normal shoulder during a telephone visit.

**Table 3:** Sample Notes for Telephone Visit With Normal Shoulder Findings

No noticeable difference between the left and right shoulders reported.
No muscle spasms noted.
No noticeable shoulder deformity noted.
No sunken areas, swelling, bruising, or red areas on the shoulder reported.
No tenderness noted during self-palpation of the chest wall, collarbone, sternoclavicular joint, acromioclavicular joint, or other parts of the shoulder.
No pain reported on rotator-cuff impingement tests modified for a telephone visit. No pain reported on provocative tests modified for a telephone visit (name each provocative test done).
No range-of-motion deficiencies reported during internal rotation, external rotation, abduction, adduction, overhead motions, or forward flexion.
No cervical pain or range-of-motion limitation while following directions over the telephone.

Shoulder Evaluation by Video

After taking a thorough history, examination of the shoulder consists of inspection, palpation, range of motion testing, strength testing, and neurovascular assessment. Inspection and range of motion is relatively straightforward during a video consultation, while strength testing, palpation, and provocative testing may require more creativity. Provocative tests may need to be modified to be self-administered and results must rely on self-reported discomfort and/or weakness. The examination should begin with a visual inspection of entire shoulder joint, arms, chest, and back, looking for any asymmetry, abrasions, swelling, changes in skin color, or striking difference between sides. Afterwards, palpation should follow. The patient should be asked to point out the precise location of the most painful area. It may be helpful to show the patient diagrams and anatomy references of the structures found within the shoulder joint. Subsequently, range of motion, strength evaluation, and neurovascular assessment should follow. 

Without a hands-on approach, range-of-motion testing is usually executed using active movements during a video encounter. Mobile applications that help measure shoulder range-of-motion have been validated to be accurate within 5° of goniometer measurements (such as Geniometer™ for Apple® devices or Geniometer Records™ for Android® devices) [26]. Household items such as a television remote control (0.5 lbs.), a can of soup or vegetables (15 oz), and a gallon of milk (4-5 lbs.) can be used as weights to help gauge strength. Lastly, provocative tests may be completed if physicians consider they may yield important data for the case. Table 4 (Figures 1A-1C and Figure 2A) provides instructions to physical examination that may be given by video, things to look for while viewing the patient on screen, and possible implications of patient responses or clinician observations [15-19,27]. Table 2 (Figures 2B-2C and Figures 3A-3C) provides questions and instructions for common provocative shoulder tests [18,20-25]. Table 5 provides sample notes for typical shoulder findings during a video visit.

**Table 4:** Shoulder Evaluation by Video – Questions and Instructions *Figure 1A; **Figure 1B; ***Figure 1C; ¤Figure 2A

What to do/say to the patient on video encounter	What to do, look for, or consider
Directly inspect the general area of and around exposed shoulders, front and back.	Watch out for asymmetry from atrophy, swelling, ecchymosis or erythema, deformity, scars, or venous distension. These may have a lot of implications that should be taken into consideration with the rest of the examination.
Inspect for any difference in height between the patient’s left and right shoulders.	Striking differences in shoulder height may suggest paraspinal muscle spasm from cervical spine pathology, nerve injury (such as spinal accessory nerve), guarding from massive rotator cuff tear, mass, acromioclavicular separation, or degenerative changes [16].
Inspect for any differences in the patient’s shoulders from a posterior view.	Striking differences in shoulder prominence from posterior view may suggest nerve injury (such as spinal accessory, dorsal scapular, or long thoracic nerve), muscle atrophy from chronic massive rotator cuff tear, sick scapular syndrome, nerve entrapment (suprascapular nerve due to paralabral cyst), brachial neuritis, iatrogenic injury, cervical radiculopathy [17].
Inspect for any, sunken, swollen, bruised, and/or red areas on patient’s shoulder?”	Sunken areas could represent atrophied areas. Swollen or bruised areas may represent contusions, fractures, or tendon ruptures. Red areas may indicate infection [17].
Inspect range of motion comparing both shoulders. Evaluate for symmetry. Begin with the patient facing camera for abduction and with arms at waist, external rotation is assessed. Forward flexion is assessed with the patient turning 90⁰ to the side, along with external/internal rotation in the shoulder closest to the camera with the shoulder abducted to 90º. Posterior reach is assessed with the patient facing away from the camera.	Range of motion (normal values) [27]: 1. Forward flexion (160–180°). 2) Extension (45°) 3. Abduction (150°) 4. External rotation (90°) 5. Internal rotation (90°) 6. Horizontal adduction (130°) 7. Posterior reach (young adults should reach tip of scapula or T7)
Ask patient to palpate the entire length of the clavicle. Show the patient (or screen-share diagrams) the general locations of surface anatomy and/or pain diagrams. Begin at sternal notch and have patient progress laterally to AC joint. Make sure to cover sternoclavicular joint, clavicle, and acromioclavicular joint. *	Pain at any of these locations suggests sternoclavicular or acromioclavicular instability, sprain, dislocation, or arthritis [15], clavicle fracture (potentially displaced if crepitus or deformity appreciated), rib fracture, supraclavicular nerve contusion, distal clavicle osteolysis [19].
Ask patient to thoroughly palpate on the frontal aspect of shoulder.	Pain in this region suggests tendinopathy or instability of long head of bicep, subscapularis tendon tear, pectoralis major rupture or strain, glenohumeral arthritis, adhesive capsulitis, anteroinferior labral tear, glenoid or proximal humerus fracture, Salter-Harris fracture in adolescents [18].
Ask patient to thoroughly palpate on the lateral aspect of shoulder.	Pain and/or weakness suggests rotator cuff tendinopathy or tear, calcific tendinitis, acromial fracture, subacromial bursitis, Salter-Harris fracture in adolescents, deltoid tear or strain (less likely) [18].
Instruct patient: “Starting with your arms hanging down at your sides, can you reach out in front of you, then upwards towards the ceiling with both arms?” **	Pain and/or weakness suggests subacromial impingement or supraspinatus pathology; cervical radiculopathy; proximal humerus or clavicle fracture; adhesive capsulitis; glenoid labrum tear; acromioclavicular joint sprain; glenohumeral arthritis; deltoid, pectoralis, or coracobrachialis tear or strain (less likely) [18].
Instruct patient: “Starting with your arms hanging down at your sides, can you reach backwards then upwards with both arms?”	Pain and/or weakness suggests adhesive capsulitis, latissimus dorsi, subscapularis tendon, or deltoid tear or strain (less common) [17].
Instruct patient: “Starting with your arms hanging down at your sides, can you reach out to your sides then upwards with both arms? Are you able to clap your hands directly above your head?” ***	Pain and/or weakness suggests supraspinatus tendon tear or tendinopathy; subacromial bursitis; cervical radiculopathy; nerve injury (such as spinal accessory, dorsal scapular, or long thoracic nerve), brachial neuritis; adhesive capsulitis; proximal humerus, acromion, or clavicle fracture; deltoid strain (less commonly) [18].
Instruct patient: “Starting with your arms hanging down at your sides, bend your elbows to 90⁰ with hands in front of you. Keeping your elbows touching your sides, can you swing your hands out to the sides away from each other?” ¤	Pain and/or weakness suggests glenohumeral arthritis, adhesive capsulitis, proximal humerus fracture [17].
Ask patient: “What tasks have you found difficult to execute due to weakness, or cause pain or range-of-motion limitation of the shoulder?” For strength testing, the clinician may also ask the patient to reproduce tasks/movements/exercises over the telephone and describe weakness and/or pain felt.	This gives the patient an opportunity to express any specific concern they may have in mind. The responses may give clues to strength problems in the shoulder. Depending on the description of the tasks causing weakness, some common pathological culprits could be the rotator cuff, biceps, or deltoid [17].
Ask patient: “Do you have any pain that runs down your arm past the elbow?”	An affirmative response is concerning for a cervical nerve root pathology [16].

**Table 5:** Sample Notes for Video Visit With Normal Shoulder Findings

Normal-looking shoulders, front and back, on inspection by video.
No areas of tenderness during self-palpation of shoulder regions, as instructed over video.
Normal over video: 1. Forward flexion (160-180°) 2. Extension (45°) 3. Abduction (150°) 4. External rotation (90°) 5. Internal rotation (90°) 6. Horizontal adduction (130°) 7. Posterior reach (young adults to tip of scapula or T7).
No weakness noted while lifting weights or pressing on nearby objects.
Negative (as adapted for video visit): 1. Apley Scratch Test 2. Speed test 3. Yergason test 4. Empty can test
5. Full can test 6. Hawkins-Kennedy test 7. Crossover test
8. O’Brien test 9. Shoulder apprehension test
Normal range of motion of cervical spine, seen over video. No pain or limitation with cervical spine flexion, extension, twisting, or side bending.
No tenderness reported on self-palpation of spinous processes or paraspinal muscles.

Radiographic considerations

Radiographic studies are a very important complement if findings from a telemedicine visit are suggestive of osseous injury or are inconclusive. Patients may have studies done locally and uploaded into a virtual imaging system or physically mailed to the treating physician to be analyzed. Imaging results may help guide management, particularly if the etiology is not completely clear based on telephone or video evaluation alone. 

Limitations of telemedicine

Despite supportive evidence, some physicians and patients may still feel hesitant to engage in telemedicine for a variety of reasons. However, a carefully planned and executed virtual medical visit may offer benefits and yield very useful information. Many physical examination tests normally performed during a shoulder evaluation may be executed by patients on themselves or a proxy examiner under the direction of a physician. All information from the patient history, physical examination (including multiple maneuvers), and imaging (if available) should be carefully considered and analyzed globally to increase the likelihood of obtaining the correct diagnosis. 

Telemedicine only works well if the technology used is at least adequate (stable internet and software, suitable cameras, speakers, and microphones, etc.) and if the patients are familiar with said technology. These are sometimes limited in isolated places or developing nations. However, with future advancements in technology, audiovisual equipment become more user-friendly, higher quality, and readily available. Attempts at early adoption of telemedicine, when suitable, will make it a satisfactory option for assessment of the shoulder and perhaps other joints as well.

## Conclusions

Telemedicine is likely to become a standard part of medical care in many places around the world. Since physical examination is particularly challenging during virtual visits, providers must carefully develop history-taking protocols to maximize the information gathering necessary to generate an accurate differential diagnosis. Musculoskeletal evaluations may be performed through telephone or video visits by asking patients to follow instructions. Musculoskeletal care is possible through telemedicine even with a joint as complex as the shoulder, and with both positive health outcomes and patient satisfaction. Beyond the COVID-19 pandemic, the ability to offer telehealth consultations is very valuable in society for a myriad of additional reasons. 
